# 3D-bioprinting a genetically inspired cartilage scaffold with GDF5-conjugated BMSC-laden hydrogel and polymer for cartilage repair

**DOI:** 10.7150/thno.38061

**Published:** 2019-09-21

**Authors:** Ye Sun, Yongqing You, Wenbo Jiang, Zanjin Zhai, Kerong Dai

**Affiliations:** 1 Shanghai Key Laboratory of Orthopedic Implants, Department of Orthopedic Surgery, Shanghai Ninth People's Hospital, Shanghai Jiao Tong University School of Medicine, Shanghai, 200011, China.; 2 Department of Nephrology, Affiliated Hospital of Nanjing Medical University, North District of Suzhou Municipal Hospital, Suzhou, China.

**Keywords:** joint dysplasia, genetics, 3d-bioprinting, hydrogel, tissue engineering

## Abstract

**Rationale:** Articular cartilage injury is extremely common in congenital joint dysplasia patients. Genetic studies have identified Growth differentiation factor 5 (GDF5) as a shared gene in joint dysplasia and OA progression across different populations. However**,** few studies have employed GDF5 in biological regeneration for articular cartilage repair.

**Methods & Results:** In the present study, we report identified genetic association between GDF5 loci and hip joint dysplasia with genome-wide association study (GWAS). GWAS and replication studies in separate populations achieved significant signals for GDF5 loci. GDF5 expression was dysregulated with allelic differences in hip cartilage of DDH and upregulated in the repaired cartilage in a rabbit cartilage defect model. GDF5 in vitro enhanced chondrogenesis and migration of bone marrow stem cells (BMSCs), GDF5 was tested in ectopic cartilage generation with BMSCs by GDF5 in nude mice in vivo. Genetically inspired, we further generated functional knee articular cartilage construct for cartilage repair by 3d-bioprinting a GDF5-conjugated BMSC-laden scaffold. GDF5-conjugated scaffold showed better cartilage repairing effects compared to control. Meanwhile, transplantation of the 3D-bioprinted GDF5-conjugated BMSC-laden scaffold in rabbit knees conferred long-term chondroprotection.

**Conclusions:** In conclusion, we report identified genetic association between GDF5 and DDH with combined GWAS and replications, which further inspired us to generate a ready-to-implant GDF5-conjugated BMSC-laden scaffold with one-step 3d-bioprinting for cartilage repair.

## Introduction

Articular cartilage injury is extremely common in congenital joint dysplasia patients 1, 2. Developmental dysplasia of hip (DDH) (OMIM # 142700) is one of the most common congenital malformations, leading to habitual hip dislocation and cartilage injury if left untreated 3, 4. DDH is characterized by reduction in acetabular coverage of the femoral head. DDH increases hip osteoarthritis (OA) risk through altered hip joint contact stress caused by a reduced weight-bearing surface area. Clinically, DDH was considered as early stage in hip OA progression and there is much overlap between DDH and OA, as they both include joint cartilage abnormality, consequent focal overstress and degradation of joint cartilage. Genetic studies have identified Growth differentiation factor 5 (GDF5) as a shared gene in joint development and OA progression across different populations 5. GDF5 belongs to the transforming growth factor beta superfamily and was strongly associated with bone and joint development 6, 7. Decreased GDF5 levels in mice contribute to osteoarthritis (OA) development by different mechanisms including altered loading and subchondral bone changes 8. Intra-articular recombinant GDF5 supplementation was reported to prevent and even reverse OA progression in rat OA model 9, 10. In OA progression, articular cartilage injury manifests early and leads to joint dysfunction, resulting in significant pain and disability of the arthritic joint 1. In a recent clinical study for DDH, the prevalence of high-grade cartilage defects could reach 40% in DDH population 11. DDH patients often present with cartilage defects on the femoral head and the acetabular surface, requiring arthroscopic procedures to repair the defects to relieve hip pain. Given the significance of GDF5 in hip joint formation, employing GDF5 in biological cartilage regeneration is promising for articular cartilage repair in DDH patients with focal cartilage defects 12.

Three-dimensional (3D)-printed scaffolds have been reported to generate different kinds of connective tissues including cartilage, bone and skeletal muscle 13-19. In cartilage engineering, 3D-printed scaffolds could provide supporting structure and mechanic strength needed for maturation of seeded chondrocytes and extra-cellular matrix (ECM) secretion in vivo 20,21. When incorporated with stem cell therapy, 3D-printed scaffolds could offer a stable environment for the recruitment of endogenous stem cells with specific peptides and the differentiation of seeded stem cells into chondrocytes in vivo 22-24. However, 3D-printed cartilage scaffolds require further cell seeding and long-term cultivation in vitro before transplantation in vivo, which lengthens the time needed for surgery and increases the infection risks in vitro 13,20,25. Hydrogel has been reported for cartilage regeneration in many studies 16,26-30. and yet it is still difficult to construct large-scale tissues with only hydrogel owing to inadequate structural integrity, mechanical stability and printability 13,31-34. Bioprinters based on jetting or extrusion methods deliver viable cells in hydrogels, biomaterials and macromolecules to generate 3D living tissues 13,35-38. Although previous studies on articular cartilage regeneration mainly focused on the seeding method to carry cells on the 3D-printed scaffold 25, we report generating genetically inspired cartilage constructs by 3D-bioprinting cell-laden Hydrogel-PCL composite scaffolds.

In the present study, we report identified genetic association between GDF5 and DDH with the largest combined genome-wide association study (GWAS), which further inspired us to explore exploiting GDF5-conjugated BMSC-laden scaffolds by 3d-bioprinting for cartilage repair.

## Results

**Figure 1:**
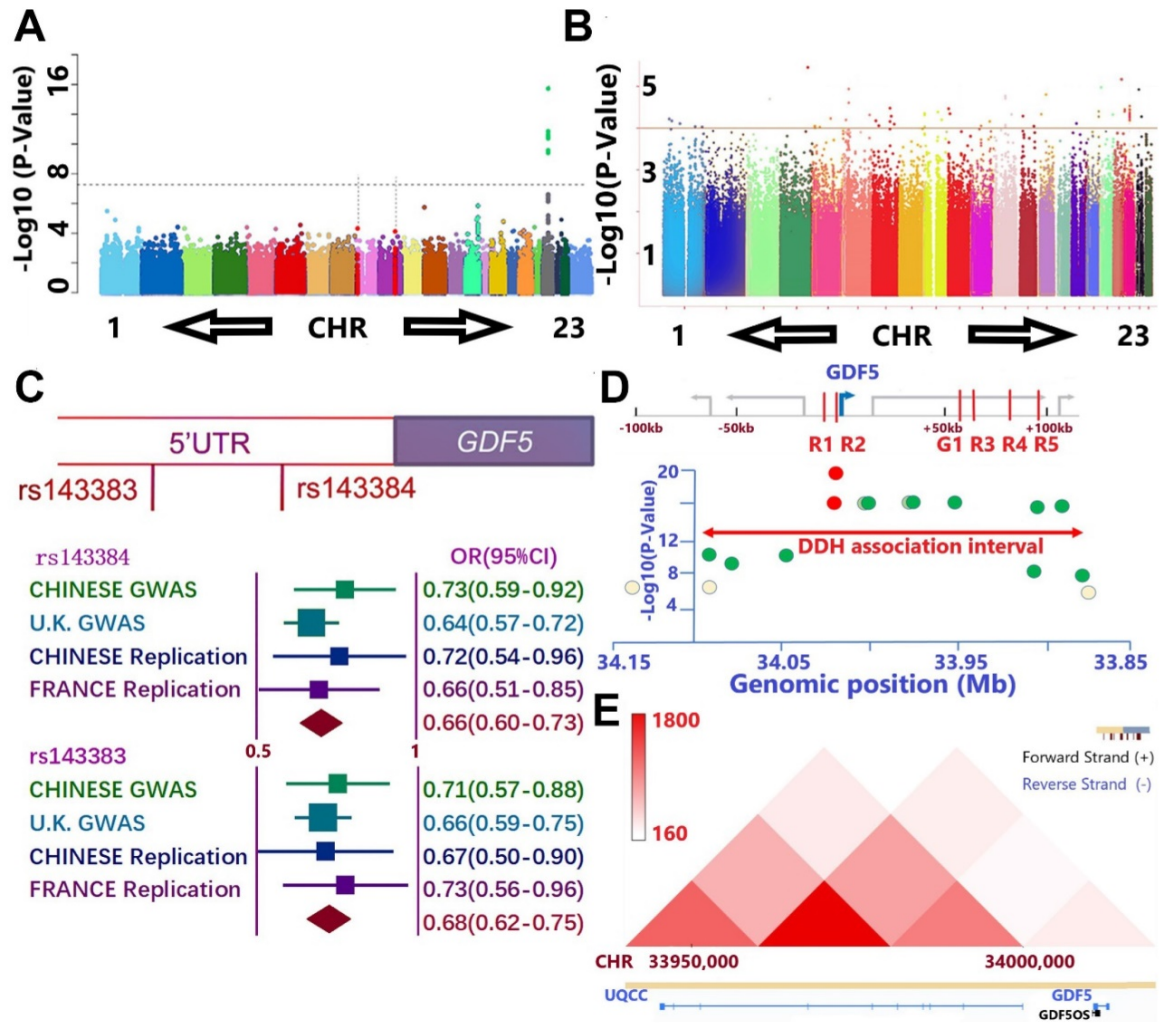
GWAS results demonstrate association between GDF5 and DDH. **A and B.** Manhattan plot of the DDH genome-wide association scan in **A)** European population and **B)** Chinese population. **A.** The dashed line indicates the genome-wide significance threshold (P =5.0 × 10-8). Green dots represent variants for which P-values reached the genome-wide significance threshold. Chromosomes X and pseudo-autosomal regions on the chromosome X are represented by number 23 and 24, respectively. **B.** No loci in Chinese GWAS reached genome-wide significance threshold and potential signals were defined as loci for which P-values were under 1 x 10-4 (dash line). **C**. A meta-analysis incorporating two functional nearing SNPs (rs143383 & rs143384) of GDF5 in osteoarthritis were derived from GWAS results, a replication study in Chinese population with 218 patients and 360 controls and achieved a significant signal in replication. (OR=0.66, 95% CI: 0.60-0.73, p=8.02E-30 for rs143384; OR=0.68, 95% CI: 0.62-0.75, p=2.68E-23 for rs143383). **D.** The genomic region linked to DDH susceptibility in human spans the regulatory enhancer architecture of GDF5 in the present study with DDH in cases vs controls. Y-axis is the -log P-value of the trait association for SNPs across the interval derived from two separate GWAS. X-axes show genomic megabase locations (bottom axis) of human sequences orthologous to reported G1, R1, R2, R3, R4, and R5 elements (red color in top axis). The highest scoring variant tested in the human study, rs143383 and rs143384 (red circle), is located in GDF5 5'UTR, immediately downstream of the R2 region. Note that significant association extends over a broad region and Some loci in the present study locate in or near the separable enhancers. **E.** Chromatin conformation capture data was acquired from human cell types to gain an understanding of the regulatory neighborhood containing GDF5 loci. Across cell types and species, we found conservation in the topologically associated domain (TAD) structure of the loci.

Separate GWAS studies of hip dysplasia in European (NJR, http://www.njrcentre.org.uk/) (**Figure** 1A) and Chinese population (**Figure** 1B) have been conducted, including totally 1156 DDH patients and 3922 controls 39,40. Potential signals in the genomic regions around GDF5 gene were identified in the discovery stage for both GWAS (**Figure** 1, C-E; Table S1 & Table S2, supporting information). Enlightened by previous reports for the functional SNPs (rs143383 & rs143384, **Figure** 1C) of GDF5 in osteoarthritis, we carried out a replication study in Chinese population with 218 patients and 360 controls and achieved a significant signal (p= 0.02 for rs143384 and p=0.007 for rs143383, **Figure** 1C). A meta-analysis incorporating the discovering GWAS, replication in Chinese and another report of the two loci in French DDH population 41 was conducted to achieve genome-wide significance for both GDF5 loci (OR=0.66, 95% CI: 0.60-0.73, p=8.02E-30 for rs143384; OR=0.68, 95% CI: 0.62-0.75, p=2.68E-23 for rs143383). (**Figure** 1C) Meta-analysis of other potential signals surrounding GDF5 gene (**Figure** 1D; Table S2, supporting information) in the discovering stage of both GWAS was also conducted to retrieve significant associations for most of the loci. Given the conservation of human GDF5 gene as well as previously reported regulatory sequence function 42-44, we next analyzed chromatin conformation capture data acquired from human cell types to gain an understanding of the broader, stable regulatory neighborhood containing GDF5 loci. Across cell types and species, we found conservation in the topologically associated domain (TAD) structure of the loci (**Figure** 1E), indicating that the large majority of regulatory interactions occur within the genomic TAD module. Some loci in the present study locate in or near separable enhancers (enhancers R1-R5 & G1, Figure 1D) within the upstream and downstream regions of GDF5 as demonstrated (**Table S2, supporting information**). The genomic region linked to DDH susceptibility in human spans the regulatory enhancer architecture of GDF5.

### GDF5 expression was dysregulated with allelic differences in hip cartilage of DDH and upregulated in the repaired cartilage in a rabbit cartilage defect model

**Figure 2:**
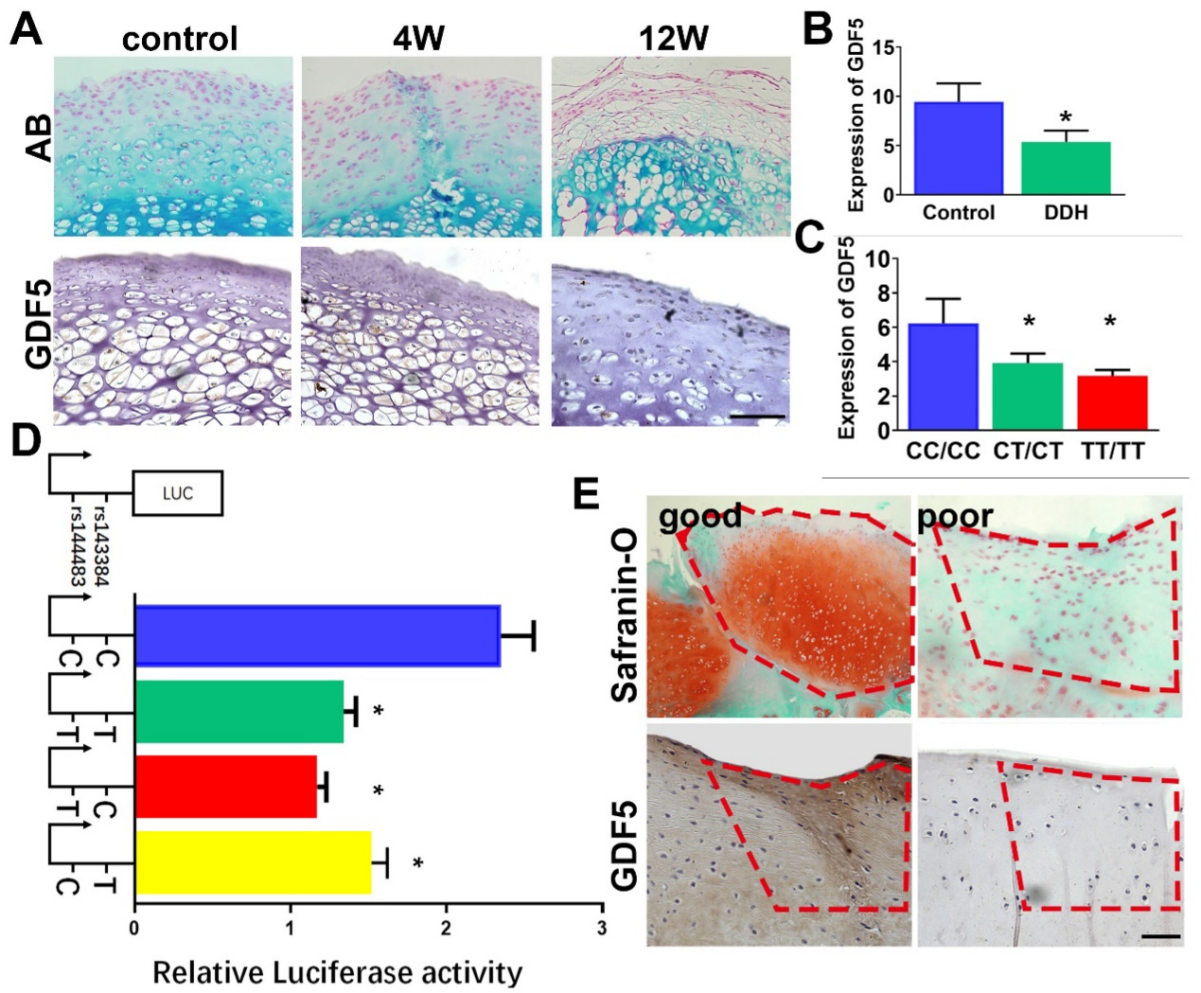
** GDF5 expression and allelic difference in DDH patients and cartilage injury. A.** GDF5 expression in hip cartilage of DDH rat hip over 12 weeks and in **B**) DDH patients(n=30) and control (n=30). Scale bar=100μm. C. Comparison of GDF5 expression among patients with different genotypes (n=12 for each genotype) are shown. *P < 0.05 between CC/CC group and other groups. **D.** Luciferase activity to indicate the allelic difference in GDF5 expression driven by haplotype (n=6 for each) of the two loci in ATDC5 cells. *P < 0.05 between C-C group and other groups. **E.** Safranin-O staining for GAG production and immunostaining for GDF5 expression in good and poor samples of neocartilage tissue in the microfracture model (n=12 in total). Scale bar=100μm. Data are presented as averages ± SD. *P < 0.05 between different groups; For two groups, statistical analysis performed using a Student's t-test while one-way analysis of variance (ANOVA) with post-hoc Tukey's B test was applied for three or more groups.

Gene expression level of GDF5 in hip cartilage samples was validated. GDF5 expression was significantly altered in DDH rat hip (**Figure** 2A) and DDH patients (**Figure** 2, B and C) respectively. In the DDH rat model, expression of GDF5 was significantly increased in hip cartilage of DDH rats samples at 4 weeks, indicating an early chondrogenic response of articular cartilage in the dysplastic hip. However, GDF5 expression was dramatically decreased in hip cartilage at 12 weeks along with much more severe arthritic changes. (**Figure** 2A) Meanwhile, expression of GDF5 was significantly decreased with significant allelic differences observed in DDH patients. (**Figure** 2, B and C) GDF5 expression among patients with different genotypes are shown. Patients with genotype CC/CC for rs143383/rs143384 were found to have a remarkably higher GDF5 expression than those with the heterogenous genotype CT/CT, and a more distinct dose-effect was observed in DDH patients with the homogenous phenotype TT/TT, showing even lower GDF5 expression. (**Figure** 2C) To verify the allelic difference in GDF5 expression by the two loci, we tested the luciferase activity driven by the haplotype of the two loci. (**Figure** 2D) In comparison, it was apparent that C-C drove greater luciferase expression than T-T, with a 40% difference seen in ATDC5 (**Figure** 2D). However, no significant difference was detected between T-T and T-C haplotypes, indicating rs143383 as the causative locus driving the luciferase expression change. Furthermore, C-T also resulted in reduced expression with a 30% difference compared to the C-C haplotype. (**Figure** 2D) These data clearly demonstrate that the two variants associated with DDH were functional and mediated decreased GDF5 expression, which partly explained the decreased GDF5 expression in DDH. Previous studies demonstrated that GDF5 was a protective factor in osteoarthritis development and exogenous GDF5 could alleviate OA progression. We analyzed the repair tissues in rabbit knee at 8 weeks after cartilage injury. The repaired tissues were immunostained for GDF5 expression and safranin-O for GAG production. Of note, we discovered significantly stronger immunostaining for GDF5 expression in neocartilage tissues with better repairing effects, indicating that GDF5 expression might enhance the repairing of cartilage injury. (**Figure** 2E) Contribution of GDF5 in chondrogenesis could also explain the reactively higher GDF5 expression at early times in dysplastic hips. Further experiments were conducted to test the potential of translating GDF5 into cartilage repairing.

### GDF5 regulated chondrogenesis of BMSCs in vitro

**Figure 3:**
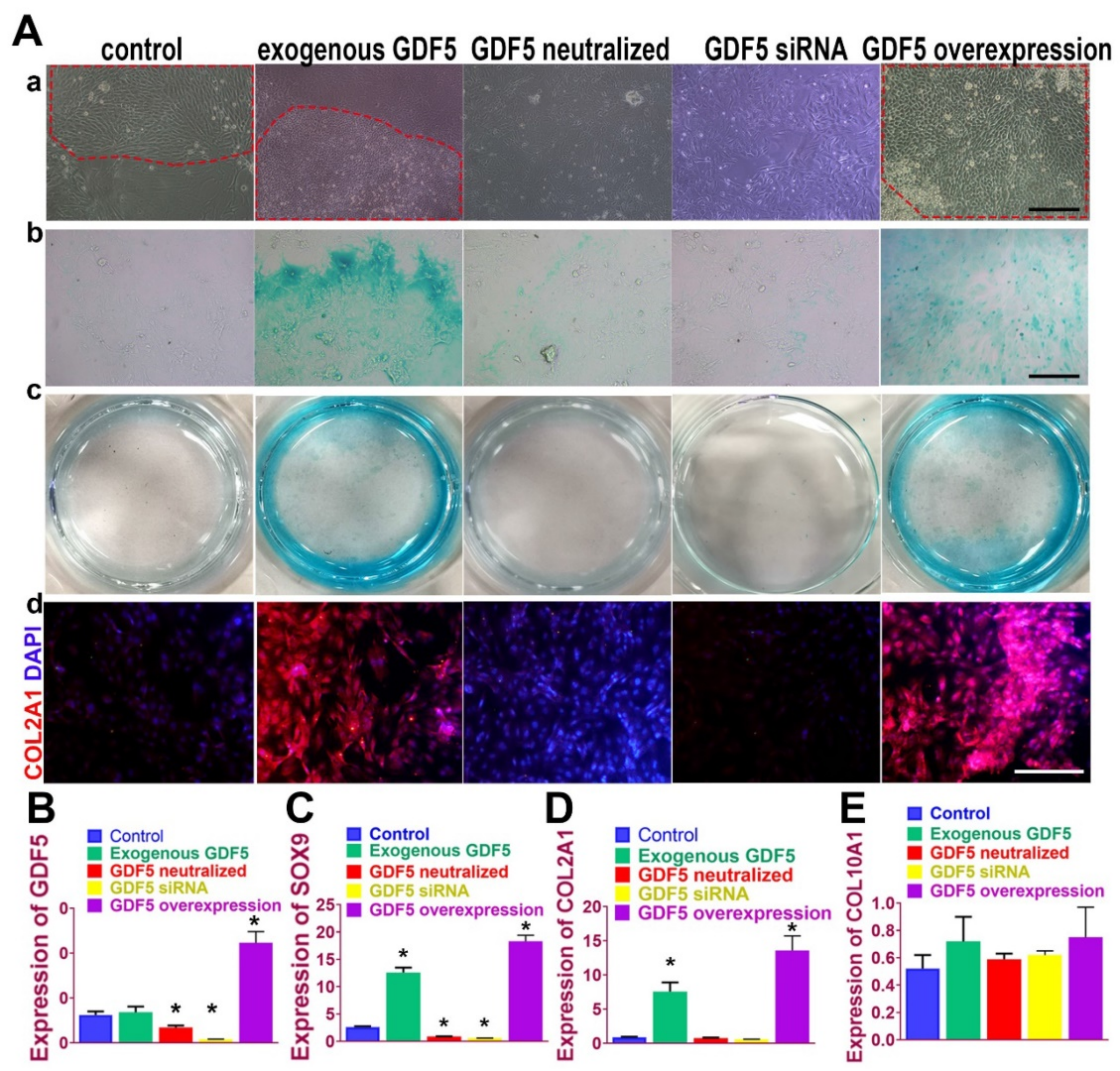
** GDF5-regulated chondrogenesis of BMSCs in vitro. A. (a)** Aggregation of rabbit BMSCs in different treatment groups at 2 weeks under light microscopy in vitro. Application of exogenous recombinant human GDF5 (100ng/ml) and GDF5 overexpression significantly induced aggregation of BMSCs (red dotted areas) and differentiation of BMSCs into articular chondrocyte-like cells. (b) alcian blue staining of BMSCs in different treatment groups under light microscopy and (c) the gross appearance of the stained cells in the culture plate. (d) Immunofluorescent assay of COL2A1 (red) expression and nucleus in different treatment groups observed under confocal microscopy. Scale bar=200μm.** B.** GDF5 expression in different treatment groups (n=6 for each) were verified using RT-PCR. *P < 0.05 between control group and other groups** C.** Expression of chondrogenic marker SOX9 in different treatment groups (n=6 for each) were verified using RT-PCR. *P < 0.05 between control group and other groups**. D.** Expression of chondrocyte marker COL2A1 in different treatment groups (n=6 for each) were verified using RT-PCR. *P < 0.05 between control group and other groups.** E.** Expression of the hypertrophic marker COL10A1 in different treatment groups (n=6 for each) were verified using RT-PCR. *P < 0.05 between control group and other groups. Data are presented as averages ± SD. One-way analysis of variance (ANOVA) with post-hoc Tukey's B test was applied.

Chondrogenic effects of GDF5 were examined on rabbit bone marrow stem cells (BMSCs) in vitro. (**Figure** 3A) Application of exogenous recombinant human GDF5 (100ng/ml) for 2 weeks in culture induced aggregation of BMSCs and differentiation of BMSCs into articular chondrocyte-like cells that synthesized glycosaminoglycan (GAG) and type II collagen. The chondrogenic effects of exogenous GDF5 was further evidenced by the its neutralization in the group with GDF-5 blocking peptide. Similar patterns for cell aggregation, GAG deposition and COL II expression were observed for the GDF5 siRNA group with depressed GDF5 expression. Compared to the control, the aggregation of BMSCs was further enhanced in the Ad-GDF5 group with GDF5 overexpression. Content of GAG in the Ad-GDF5 group, similar to the exogenous GDF5 group, was significantly higher with much stronger alcian blue staining than that of the control group (Figure 3A). Immunofluorescent assay showed significant difference in COL II expression between Ad-GDF5 and control. Chondrocytes generated under the several conditions were next analyzed with RT-PCR (**Figure** 3, B-E; **Figure S1**, supporting information) for expression of genes expressed by both articular and hypertrophic chondrocytes (SOX9, COL1A1, COL2A1 and COL10A1). Compared to the control, exogenous GDF5 treatment and Ad-GDF5 group with GDF5 overexpression both led to significant chondrogenic effects evidenced by SOX9 and COL2A1 expression. However, no significant change was observed for COL1A1 and COL10A1 expression among different groups, indicating that mainly non-hypertrophic articular chondrocytes with hyaline cartilage phenotype were generated by GDF5 supplementation.

### Ectopic cartilage generation with BMSCs by GDF5 in nude mice in Vivo

**Figure 4:**
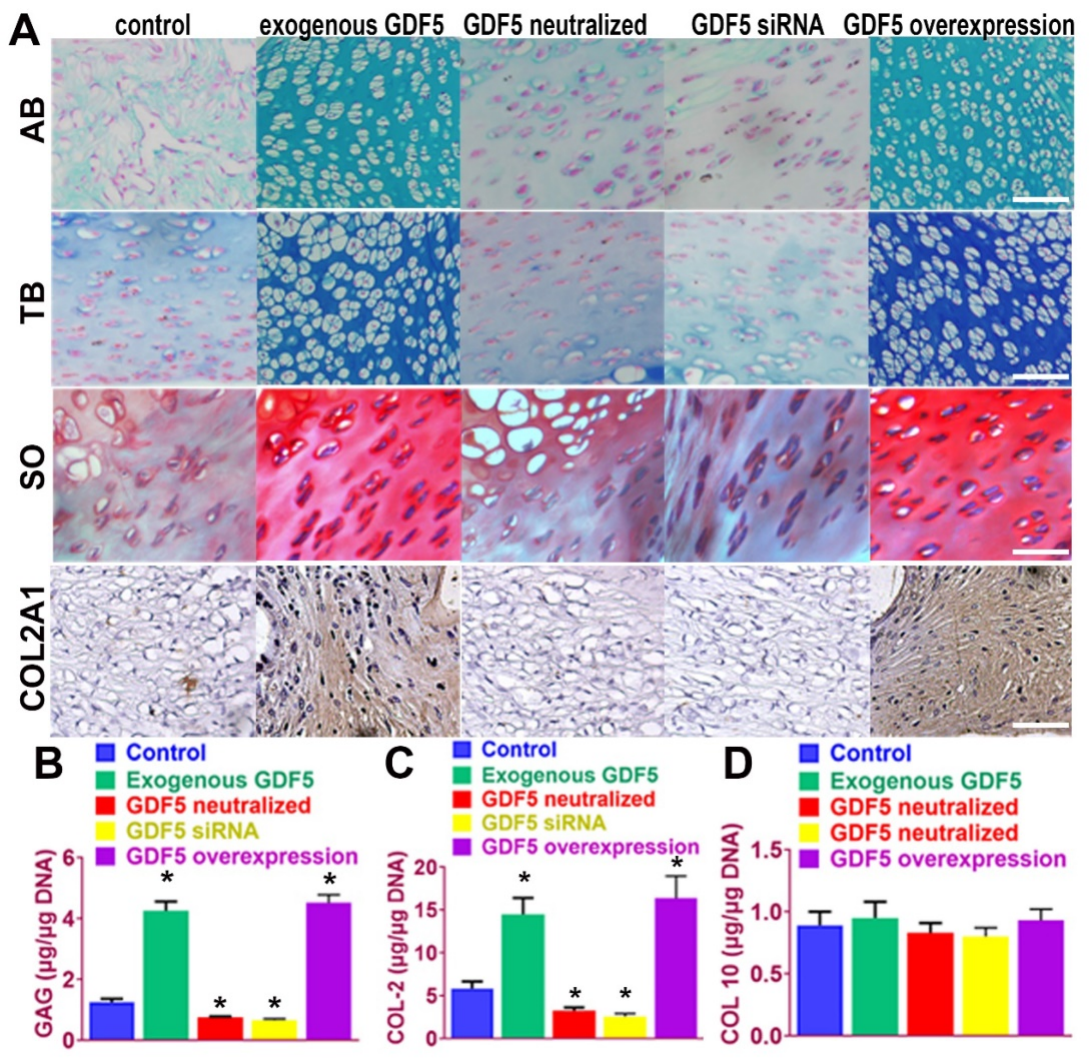
** Ectopic cartilage generation with BMSCs by GDF5 in nude mice in vivo A**. The chondrogenic ability of induced BMSC cells in different groups to generate ectopic cartilage in vivo by subcutaneously injecting induced cells into the dorsal flanks of nude mice. Histological examination of the injected sites was conducted for GAG production with metachromatic staining with alcian blue (AB, first row), toluidine blue (TB, second row) and safranin-O (SO, third row) and immunostaining of type II collagen expression (forth row). Scale bar=100μm. **B.** GAG quantification in generated ectopic cartilage tissues in different groups (n=6 for each). *P < 0.05 between control group and other groups. **C.** quantification of COL II and D) COL X expression in generated ectopic cartilage tissues in different groups (n=6 for each). *P < 0.05 between control group and other groups. Data are presented as averages ± SD. One-way analysis of variance (ANOVA) with post-hoc Tukey's B test was applied.

We next examined the potential of induced BMSC cells to generate ectopic cartilage in vivo by subcutaneously injecting induced cells and into the dorsal flanks of nude mice (**Figure** 4). Four weeks later, histological examination of the injected sites was conducted for GAG production and type II collagen expression. (**Figure** 4A) Among the five cell lines, BMSCs supplemented with exogenous GDF5 and Ad-GDF5 BMSCs with GDF5 overexpression produced substantial amounts of GAG in generated cartilage tissues, as indicated by strong metachromatic staining with alcian blue, toluidine blue and safranin-O (**Figure** 4A, first to third rows). Lacuna formation, a typical sign of cartilage formation, was also observed. Immunohistochemical analysis showed that the cartilage generated by injection of Ad-GDF5 BMSCs and BMSCs with exogenous GDF5 treatment in the nude mice deposited rich GAGs (**Figure** 4B) and expressed abundant type II collagen (**Figure** 4A, forth row; 4C). No significant difference was detected for COL 10 expression among different groups (**Figure** 4D), confirming that hyaline cartilage was generated. These results suggest that BMSCs with exogenous GDF5-treatment or genetically modified BMSCs with GDF5 overexpression could both generate ectopic cartilaginous tissue in vivo.

### GDF5 enhanced migration of BMSCs both in transwell assay and in a fabricated cartilage scaffold

**Figure 5:**
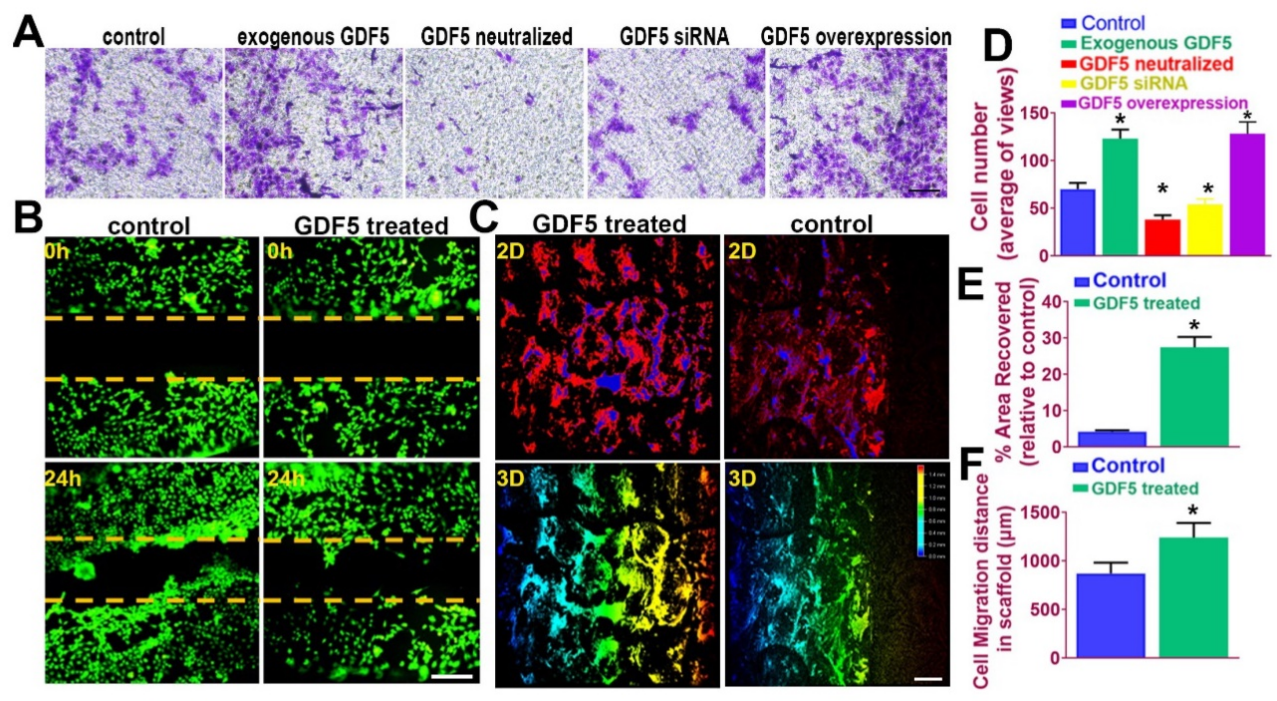
** GDF5 enhanced migration of BMSCs both in transwell assay and in a fabricated cartilage scaffold. A. T**he effects of GDF5 on BMSC migration observed under light microscopy in different treatment groups with transwell assay. Scale bar=100μm. **B.** Scratch assay to demonstrate the GDF5-induced wound healing capability of BMSCs in hydrogel over 24 hours. BMSCs were stained with calcein dye. Scale bar=100μm.** C.** Migration assay of BMSCs over 2 weeks in the scaffolds under confocal microscopy in the GDF5-treated group and the control group. The total migration distance in height axis of the confocal microscopy for examination was 1.5mm. First row, 2D view; Second row, 3D view. Scale bar=200μm** D.** The number of migrated BMSC in different treatment groups (n=6 for each) in transwell assay. *P < 0.05 between control group and other groups. **E.** Covered areas in the scratched area of the hydrogel over 24 hours in the scratch test for both groups (n=6 for each). **F.** Comparison of the migration distance for BMSCs in the scaffolds over 2 weeks (n=6 for each). Data are presented as averages ± SD. *P < 0.05 between different groups; For two groups, statistical analysis performed using a Student's t-test while one-way analysis of variance (ANOVA) with post-hoc Tukey's B test was applied for three or more groups.

It is acknowledged that recruitment and migration of MSCs to the defect site in vivo is beneficial for cartilage repair and leads to better lesion healing 45. In this case, we examined the effects of GDF5 on BMSC migration in different treatment groups by transwell assay. (**Figure** 5A) The number of migrated BMSCs in the exogenous GDF5 group and the GDF5 overexpression group with Ad-GDF5 BMSCs were significantly greater compared to that of the control group in transwell analysis (**Figure** 5, A and D). To examine the cartilage healing potential of GDF5-conjugated BMSC-laden hydrogel, a scratch assay was conducted for BMSCs embedded in the composite hydrogel (Table S3, supporting information). GDF5 supplementation significantly increased hydrogel-embedded BMSC migration in the scratch assay with much more covered areas in the scratched area over 24 hours (**Figure** 5, B and E). Furthermore, we used porous poly(ε-caprolactone (PCL) cartilage scaffolds (details of the PCL scaffold fabrication were in **Figure** 7) to examine the migration of BMSC in the scaffolds by placing the scaffolds atop monolayer-cultured BMSCs. The scaffolds were incubated and observed for 2 weeks in vitro. (**Figure** 5C) Compared to the control group, GDF5-treated scaffolds showed significantly longer migration distance for BMSCs within. (**Figure** 5, C and F) Higher coverage by migrated BMSCs surrounding the scaffolds were also observed in the GDF5 treated group than that of the control group. (**Figure** 5F) These results confirmed that the GDF5 treated scaffold showed better MSC recruitment and migration than the control. Therefore, we assume the GDF5-conjugated BMSC-laden scaffold could significantly enhance chondrogenesis of loaded BMSCs in vivo and improve endogenous MSC migration toward the scaffold, providing a good prospect and a powerful tool for cartilage repair.

### Fabrication of GDF5-conjugated BMSC-laden scaffold for cartilage repair

**Figure 6:**
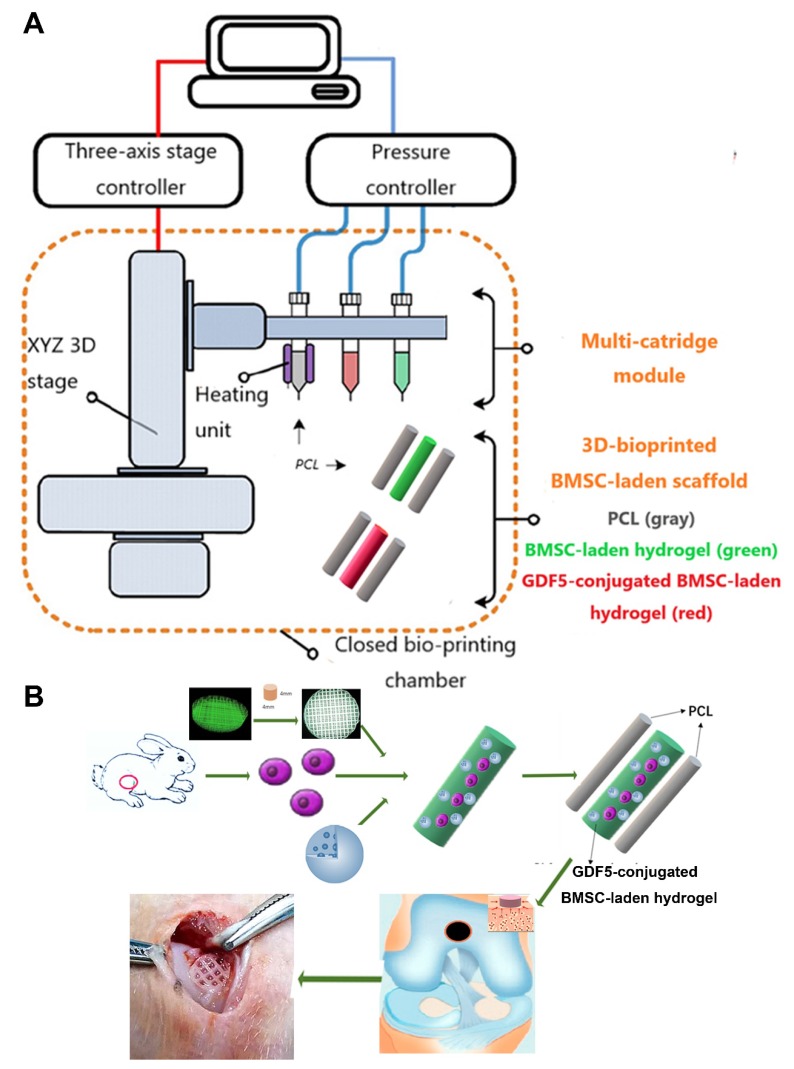
** Schematic Illustration of t**he** printing system and study design. A.** The printing system OPUS resides in a closed acrylic chamber consisting of a 3-axis stage controller for the 3D motion and the dispensing module including multiple cartridges and pneumatic pressure controller. In the designed cartilage construct, GDF5-conjugated μS (red) and empty μS (green) were mixed in the BMSC-laden hydrogel respectively and printed into the microchannels between PCL fibers with different syringes in the scaffolds in different groups. **B.** Schematic Illustration of the study design with 3D-bioprinted GDF5-conjugated BMSC-laden hydrogel-polymer composite constructs for articular cartilage regeneration in rabbits.

**Figure 7:**
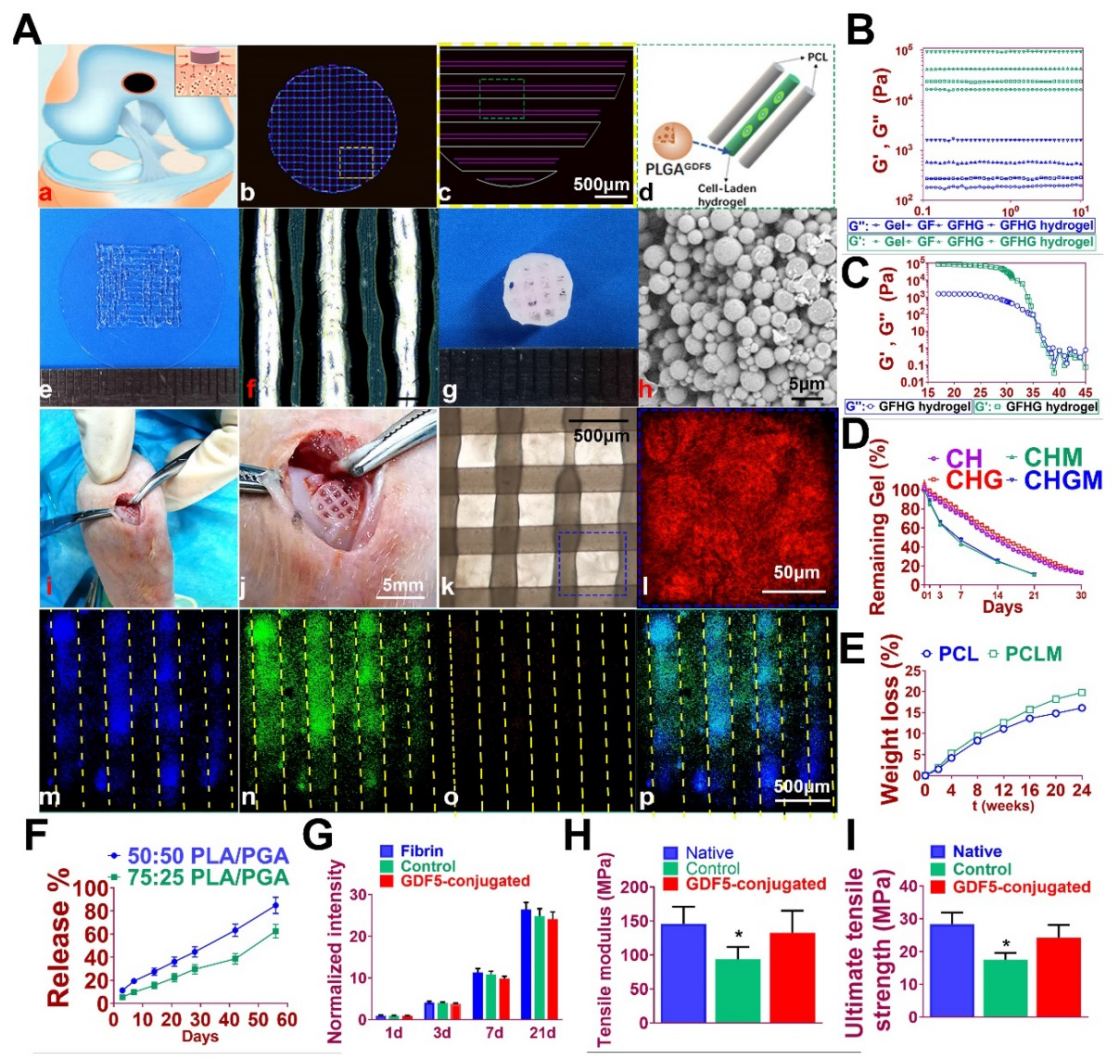
** Fabrication of GDF5-conjugated BMSC-laden scaffold for cartilage repair. A.** 3D-bioprinted cartilage scaffold for implantation. (a) Schematic Illustration of constructed scaffolds for cartilage repair in rabbit knee. (b) 3D CAD of each layer of the cartilage scaffold and (c) the dispensing path (yellow box outlined in b) of (d) aligned PCL and hydrogel (green box outline in c). GDF5-conjugated BMSC-laden hydrogel was dispensed into the space between PCL fibers. (e) Gross appearance of GDF5-conjugated BMSC-laden hydrogel printed with OPUS. (f) Hydrogel was further observed under light microscopy. (g) Gross appearance of the cartilage scaffold with GDF5-conjugated BMSC-laden hydrogel and PCL as supporting structure. (h) SEM images of GDF5-conjugated PLGA μS. (i) Implantation process of the cartilage scaffold into the defect site in a rabbit knee. (j) Higher resolution image of the implanted scaffold in (i). (k) The alignment of PCL and hydrogel in the scaffold under microscopy. (l) Higher resolution image of the blue box area outlined in (k). (m to p) Minimal toxicity and distribution PLGA μS in BMSC-laden hydrogel in the scaffolds. (m) Fluorophore-conjugated rhodamine was encapsulated into PLGA μS and delivered to the hydrogel in the printed scaffolds. (n) At day 7, live BMSCs and (o) dead BMSCs in the PLGA-conjugated hydrogel printed between the PCL fibers were demonstrated with live/dead assay and observed under confocal microscope. (p) Merged image for (m to o). **B.** Mechanical spectra of different component (gel: gelatin; GF: gelatin + fibrinogen; GFHG: gelatin + fibrinogen + hyaluronic acid + glycerol) and the cross-linked hydrogel (GFHG hydrogel) measured at 17 °C. **C.** Dynamic thermal rheological observations of the cross-linkage of GFHG. **D.** Degradation rate of BMSC-laden hydrogel and **E.** PCL in vitro and in vivo. CH: BMSC cell-laden hydrogel: CHG: BMSC cell-laden hydrogel with conjugated GDF5. CHM and CHGM: CH and CHG assessed in nude mice.** F.** Released GDF5 concentration from PLGA μS was measured using enzyme-linked immunosorbent assay (ELISA) kits. Spheres showed controlled release of GDF5 that sustained over 60 days in vitro. **G.** Cell proliferation in the cartilage scaffolds. To determine cell proliferation in the scaffolds, we examined survival of BMSCs in the scaffolds compared to BMSCs cultured in fibrin through 21 days post printing with AlamarBlue assay kit. H. Biomechanical properties of the in vitro cartilage construct, including bulk tensile modulus and I) UTS after 12 weeks of culture. *P < 0.05 between the native or the GDF5-conjugated group and control group. All data are means ± SD (n = 6) and were analyzed by two-way ANOVA with Tukey's test.

GDF5-conjugated BMSC-laden scaffold was constructed for cartilage repair in rabbit knee (Figure 6). Rabbit BMSCs were derived for cell delivery in hydrogel. (Figure 7A; Table S3, supporting information) Cell carrier hydrogel was produced with a mixture of gelatin, fibrinogen, HA and glycerol.** (Figure 7, B and C; Table S3, supporting information)** Gelatin was used for its thermo-sensitive properties (liquid above 37 °C and solid below 25 °C). Fibrinogen provides stability to the gel and a better microenvironment favorable to cell adhesion and proliferation. HA and glycerol could enhance the path uniformity in dispensing and prevent nozzle clogging at low temperature. This allowed for the creation and reservation of microchannels made between cell-laden hydrogel and PCL patterns, which would further allow for the diffusion of nutrient and oxygen into the printed cartilage constructs. Dynamic mechanical analysis was performed for the composite hydrogel **(Figure 7, B and C),** Storage modulus G′ and the loss modulus G′′ are presented **(Figure 7B),** exhibiting evident plateau in the frequency range investigated. Thermal scanning rheological observation was also made for the gel** (Figure 7C).** Obtained hydrogel showed high G′ values at low temperatures, but the storage and loss modulus decreased on heating with a crossover of G′′ and G′ at temperature of 35℃. This temperature indicates the transition from an elastic network formation to a solution for the gel. Degradation of the composite hydrogel and PCL was also explored in vitro and in vivo in nude mice **(Figure 7, D and E).** PCL degraded much slower than the composite hydrogel, providing structural integrity for the focal repaired cartilage and mechanic support in weight bearing as the scaffold degrades. 3D cartilage structures were produced by placing together GDF5-conjugated BMSC-laden hydrogel and PCL polymer (~100 μm diameter for hydrogel and ~200 μm diameter for PCL) to construct a composite cartilage scaffold using organ printing united system (OPUS, Novaprint). Briefly, PCL was molten to fabricate the supporting structure for the cartilage scaffold while BMSC-laden hydrogel encapsulating poly (lactic-co-glycolic acid) (PLGA) microparticles carrying GDF5 were bioprinted into the microchannels (300 μm) between PCL fibers from a different syringe (**Figure** 6, A and B). A computer-generated 3D tissue model was converted into a motion program that operates and guides the dispensing nozzles to take defined paths for delivery of hydrogels and polymers. (**Figure** 7A, a to c) GDF5-conjugated BMSC-laden hydrogel was dispensed into the space between PCL fibers. After printing, the printed cartilage constructs were cross-linked by addition of thrombin to further maintain the shape fidelity of the hydrogel**.** PLGA (50:50 PLA/PGA) microspheres(μS) encapsulating GDF5 was mixed to deliver GDF5 in hydrogel. (**Figure** 7A, d) Released GDF5 concentration from PLGA μS was measured using enzyme-linked immunosorbent assay (ELISA) kits. Spheres showed controlled release of GDF5 that sustained over 60 days in vitro (**Figure** 7F). The GDF5-conjugated BMSC-laden hydrogel showed nice printability as demonstrated (**Figure** 7A, e to g) and scanning electron microscope (SEM) images of most PLGA μS demonstrated a less than 5μm diameter. (**Figure** 7A, h) The printed scaffold showed delicate and orderly hydrogel-PCL alignment under light microscope and fits right into the defect site in transplantation. (**Figure** 7A, i to l) To validate μS distribution in MSC cell-laden hydrogel, fluorophore-conjugated rhodamine was encapsulated in to PLGA μS and delivered to the hydrogel. At day 7, rhodamine-conjugated PLGA μS showed well-proportioned distribution in the cartilage scaffold as well as minor cell toxicity in the hydrogel printed between the PCL fibers under confocal microscope (**Figure** 7A, m to p). Cell viability in the scaffolds was further examined for survival of BMSCs at 21 days post printing. Live/dead cell assays showed ≥95% cell viability on day 0, which was maintained through 21 days with cell proliferation rate similar to BMSCs cultured in fibrin (**Figure** 7G). Biomechanical properties of the 3D-bioprinted cartilage constructs were assessed after 12-week cultivation in vitro before in vivo implantation. The GDF5-conjugated scaffolds showed higher tensile modulus and greater ultimate tensile strength (UTS) than scaffolds in the control group (**Figure** 7, H and I), reaching the values for the native cartilage with no significant differences. These results indicate that our GDF5-conjugated BMSC-laden hydrogel-PCL composite cartilage scaffold not only restored biomechanical properties of the native cartilage, but also maintained cell viability after printing and provided a favorable microenvironment for BMSC proliferation and further differentiation into chondrocytes in vitro.

### GDF5-conjugated BMSC-laden Scaffold implantation demonstrated better repairing effect of cartilage in rabbit knee cartilage defect model in vivo

**Figure 8:**
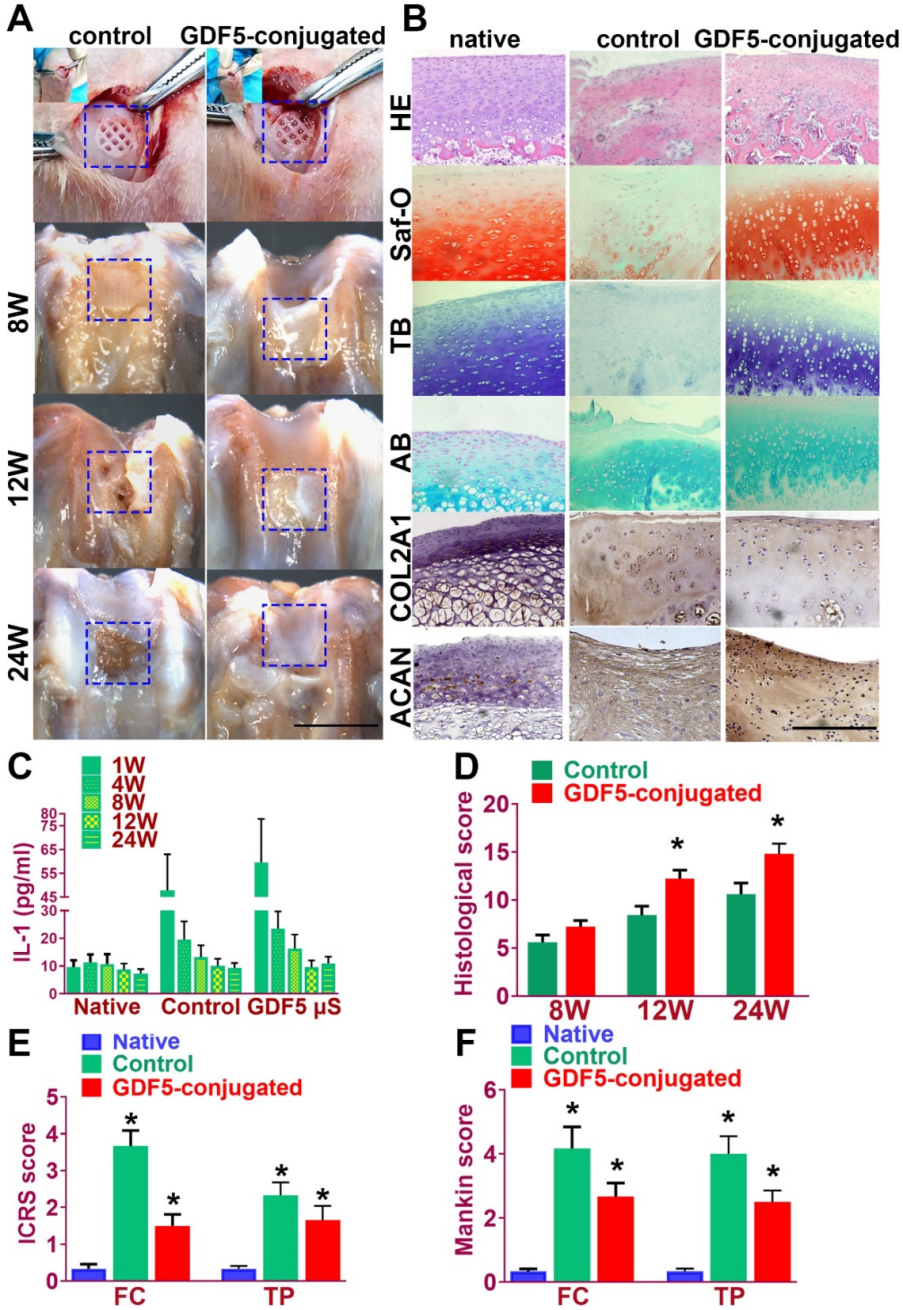
** GDF5-conjugated BMSC-laden Scaffold implantation demonstrated better repairing effect of cartilage in rabbit knee cartilage defect model in vivo. A.** Rabbits (n=6 for each group) were used as animal models to evaluate the knee repair capacity of the scaffolds. Full-thickness cartilage defect was created in the rabbit knee and the scaffolds were implanted into the defect site (first row) for cartilage tissue regeneration over 24 weeks. Scale bar=5mm. **B.** Histological analysis was conducted of the repaired cartilage tissue sections with H&E (first row), safranin-O (second row), toluidine blue (third row) and alcian blue (forth row) staining to evaluate cartilage regeneration by different scaffolds compared to the native cartilage. Immunohistochemical staining of cartilage markers ACAN and Collagen II (fifth and sixth rows) for chondrocyte phenotype was conducted in the generated cartilage tissue sections in different groups compared to the native cartilage. (fifth and sixth rows) Scale bar=500μm. **C.** Examination of intra-articular inflammatory response in the joint fluid was conducted with quantification of IL-1 concentration using ELISA kit. *P < 0.05 between 1W group and other groups at the same time point **D.** Histological grading of the repaired cartilage for the GDF5-conjugated scaffolds compared to the scaffolds in the control group over 24 weeks. *P < 0.05 between control group and the GDF5-conjugated group. **E.** ICRS histological score of articular cartilage in the femoral condyle (FC) and tibial plateau (TP) in both groups with scaffold implantation. *P < 0.05 between native group and other groups. **F.** Mankin histological score of articular cartilage in the FC and TP in both groups with scaffold implantation compared to the native cartilage with no implantation surgery. *P < 0.05 between native group and other groups. Data are presented as averages ± SD; For two groups, statistical analysis performed using a Student's t-test while one-way analysis of variance (ANOVA) with post-hoc Tukey's B test was applied for three or more groups.

Rabbits were used to evaluate the knee repair capacity of the scaffolds. As shown in **Figure** 8A, full-thickness cartilage defect was created in the rabbit knee. The scaffold was implanted into the defect site to test for cartilage tissue regeneration. Cartilage repair with GDF5-conjugated BMSC-laden scaffold showed much better gross appearance at 8, 12 and 24 weeks compared to the control scaffolds with only BMSCs (**Figure** 8A). At 24 weeks, H&E staining was used to show the integrity of formed neocartilage tissue. Neo-cartilage in the GDF5-conjugated group showed more similar appearance to normal cartilage than the control groups (**Figure** 8B, first row). Histomorphological analysis with safranin-O, toluidine blue and alcian blue staining for GAG was used to evaluate generated cartilage by the scaffolds compared to native cartilage. As shown in **Figure** 8B, when the defect was treated with the GDF5-conjugated scaffold, fully hyaline-like cartilage was regenerated, as evidenced by intense safranin-O, toluidine blue and alcian blue staining for GAG (**figure** 8B, second to forth row) and better cell filling in H&E staining (**Figure** 8B, first row).

Immunohistochemical staining of cartilage markers (ACAN and Collagen II) for chondrocyte phenotype was conducted in the generated cartilage tissue sections in different groups (**Figure** 8B, fifth and sixth rows). Compared to the control group, stronger intensity in ACAN and COL II staining, which resembles the native cartilage, was observed in the generated cartilage in the GDF-5 conjugated BMSC-laden scaffold group, indicating successful reconstruction of articular cartilage with abundant ECM deposition (**Figure** 8B). The chondroprotective effects of the scaffolds were also tested in vivo (**Figure** 8, C-F). Examination of intra-articular inflammatory response in the joint fluid was conducted with quantification of IL-1 concentration. Compared to the non-operative native group, scaffold implantation in both groups initiated a transient increase during the acute phase post implantation (one week). The concentration of IL-1 in the two scaffold groups began to decline at 4 weeks and maintained at a relatively low level during the 24 weeks of cartilage repair process. No statistically significant difference was observed for the two groups with scaffolds at 24 weeks compared to the non-operative native group (**Figure** 8C). Histological grading of the repaired cartilage demonstrated a better repairing effects of GDF5-conjugated scaffolds compared to the scaffolds in the control group over 24 weeks (**Figure** 8D). However, articular cartilage in both groups with scaffold implantation showed elevated ICRS and Mankin histological score compared to the native cartilage with no implantation surgery. Compared to the control group, the GDF5-conjugated scaffolds showed better chondroprotective effects with a significantly lower histological grading in the femoral condyle (FC) and tibial plateau (TP) over the 24 weeks in vivo (**Figure** 8, E and F). In summary, these results indicated that, compared to the scaffolds with only BMSCs loaded, the GDF5-conjugated BMSC-laden scaffold not only showed better cartilage repairing effects, but better maintained joint function with low intra-articular inflammatory response after transplantation.

## Discussion

In the present study, we report genome-wide association between GDF5 gene and DDH with the largest combined GWAS and follow-up replications in multiple populations, identifying multiple loci and genomic regions spanning GDF5 regulatory elements in association with DDH. Inspired by the genetic association between joint dysplasia and GDF5, we have further explored exploiting GDF5-conjugated BMSC-laden scaffolds by 3d-bioprinting for cartilage repair. GDF5 is recognized as one of the most important genes affecting skeletal development. Although several SNPs in GDF5 gene were reported for bone and joint system 46, only the genetic deficit of rs143383 and rs143384 was uniquely demonstrated to mediate osteoarthritis 47. Reconstruction experiments have shown that the derived “T” risk alleles at rs143383 and rs143384 reduce the quantitative levels of GDF5 expression when transfected with reporter genes into tissue cells in vitro 47. Deep sequencing of GDF5 in patients with severe primary OA from three populations (UK, Spanish, Greek) identified no rare variants in all cohorts 48, further implicating the significance of the regulatory regions for GDF5 in joint morphogenesis. Regulatory elements controlling GDF5 expression in synovial joints have been identified in previous studies 42,44, concluding that modular GDF5 enhancers controlled development of different joints including heads, shoulders, elbows, knees and toes in the vertebrate skeleton. The present study identified several DDH susceptibility loci in these enhancers and further studies are still needed to clarify the specific influence of these loci and the corresponding enhancers on hip joint formation and morphogenesis.

Genetically inspired by hip joint dysplasia, we further generated functional knee articular cartilage constructs for cartilage repair by 3d-bioprinting a GDF5-conjugated BMSC-laden scaffold. Chondrogenic effects of GDF5 on BMSC and adipose-derived stem cells in vitro have been explored in previous studies 49-51. Feng et al 50 induced GDF5 expression with GDF5 adenovirus and identified comparable chondrogenic effects to exogenous GDF5 supplementation. Murphy et al 51 yielded mechanically robust cartilage rich in collagen II and GAGs in both BMSC and ADSC with TGF‐β1, GDF‐5, and BMP‐2 stimulation for 4 weeks in vitro. In a recent study, Zhu et al 52 delivered GDF5 and ADSCs into intervertebral spaces for disc degeneration treatment in rats and retrieved promising outcomes for GDF5 in tissue engineering. The treatment effects of GDF5 could also be attributed to protective effects of GDF5 on ECM and maintaining of chondrocyte phenotype since the nucleus pulposus consisted of mainly chondrocytes and ECM in the intervertebral disc, which was quite similar to the native articular cartilage. However, cartilage repairing was more challenging in comparison due to the structural strength needed and no previous studies have incorporated GDF5 in 3d-bioprinted constructs for articular cartilage regeneration. In the present study, cartilage constructs with structural strength and integrity ready for surgical implantation were created by sequentially co-printing GDF-conjugated BMSC-laden hydrogel with synthetic PCL polymer. The hydrogel allowed well-proportioned distribution of BMSCs and GDF5-conjugated μS, and thus protects BMSC viability and promotes GDF5-induced differentiation and in the scaffolds. Meanwhile, the PCL scaffolding provides adequate mechanical support and architectural integrity to offer a stable microenvironment for BMSCs within the hydrogel to differentiate and form tissue within secreted cartilage matrix as the hydrogel degrades. PCL is biocompatible and flexible with a low melting temperature around 60 °C, which could minimize cell damage from heat after its rapid cooling after printing and enabled its co-printing with cell-laden hydrogel 53. PCL also showed a relatively long degradation time (~1 to 2 years), which provides long-term structural stability for the repaired cartilage 54. In contrast, materials with more rapid degradation often generate byproducts and cause structural and dimensional deformation of the scaffolds 55-58. Lineage-tracing studies have provided compelling evidence that articular chondrocytes derive from GDF5-lineage interzone cells in regions of the condensing chondrogenic mesenchyme 59-62, similar to our observed condensation of BMSCs in culture and in the small compartments within surrounding PCL fibers as supporting structure. In the presence of GDF5, these BMSCs would further differentiate into articular chondrocytes that express markers for the native cartilage 63. GDF5-conjugated scaffold could also enhance the migration of endogenous GDF5-traced chondroprogenitors from the synovium and interzone, contributing to the healing of repaired cartilage. The defect model we conducted in rabbits was a 4x4x4mm cartilage defect and it was quite a large defect for a rabbit knee. Articular cartilage has limited potential to self-generate focal defects larger than 1mm. To ensure a successful defect model and yet enable normal and comparable mobilization post-modeling, we did not include an untreated group. However, an untreated control group would have been good for comparison to the groups with implanted scaffolds. For translation, we envision a GDF5-conjugated 3d-bioprinted human-scale cartilage scaffold ready to implant in a surgery where the surgeons could incorporate surgery and 3D-bioprinting by performing replacing the damaged or degenerated joint cartilage with 3d-bioprinted cartilage scaffolds using mini-invasive arthroscopy procedures 24,64.

## Conclusions

In conclusion, we report identified genetic association between GDF5 and DDH with combined GWAS and replications, which further inspired us to generate a ready-to implant GDF5-conjugated BMSC-laden scaffold with one-step 3d-bioprinting for cartilage repair.

## Abbreviations

3D: Three-dimensional
ACAN: aggrecan
BMSC: Bone marrow stromal cell
DDH: Developmental dysplasia of the hip
ECM: extra-cellular matrix
ELISA: enzyme-linked immunosorbent assay
GAG: glycosaminoglycan
FC: femoral condyle
GDF5: Growth differentiation factor 5
GWAS: genome-wide association study
OA: osteoarthritis
PCL: poly(ε-caprolactone)
PLGA: poly (lactic-co-glycolic acid)
SEM: scanning electron microscope
TAD: topologically associated domain
TP: tibial plateau
UTS: ultimate tension modulus
