# Supplementary material for: 3D-bioprinting a genetically inspired cartilage scaffold with GDF5-conjugated BMSC-laden hydrogel and polymer for cartilage repair
Source: Theranostics. 2019 Sep 21;9(23):6949–61. doi: 10.7150/thno.38061 (PMC6815949; doi:10.7150/thno.38061)
Supplement: Supplementary file 1 — Supplementary figures and tables. [file thnov09p6949s1.pdf]

Supporting Information

3D-bioprinting a genetically inspired cartilage scaffold with GDF5-conjugated BMSC-laden hydrogel and polymer for cartilage repair

Ye Sun[1,#,*], Yongqing You[2,#],Wenbo Jiang[1], Zanjin Zhai[1], Kerong Dai[1,*]

Materials & Methods

Patients

Separate GWAS studies of hip dysplasia in the NJR and Chinese population have been conducted, including 1156 hip dysplasia patients and 3922 controls.[1, 2] Genotype data of the promising loci in GDF5 was extracted from the GWAS studies. A further replication study for the two potential loci (rs143383, rs143384) with 218 DDH cases and 360 controls was conducted in Chinese Han population. A meta-analysis incorporating the discovering GWAS, replication in Chinese and another report of the two loci in French DDH population[3] was conducted to achieve genome-wide significance for the two GDF5 loci. Bilateral chi square tests were conducted to determine the significance of differences in allelic frequencies and P < 5 x 10-8 was considered to be statistically significant.

DDH patients were consecutively recruited from the department of orthopedics of Ninth Hospital, which is affiliated with Shanghai Jiao Tong University. Controls were recruited from physical examination center in the Ninth Hospital. DDH was diagnosed according to clinical criteria and radiographic evidence. All controls were confirmed to have no symptom or history of DDH. Subjects with any genetic disease except DDH were excluded. All the subjects were

Han Chinese living in or around Shanghai. The study was approved by the Ninth Hospital and the medical school of Shanghai Jiao Tong University Ethics Committee and informed consents were obtained from all patients and controls. All methods were performed in accordance with the relevant guidelines and regulations

Genotyping of targeted locus

According to the manufacture's protocol, the DNA of all the subjects was extracted either from the buccal swabs using the DNA IQ System (Promega, Madison, WI) or peripheral blood using the NucleoSpin Blood QuickPure Kit (Macherey-Nagel GmbH & Co. KG, Düren, German). All the samples were genotyped with Taqman assay. The sample was genotyped by uninformed laboratory personnel. Genotyping, data input and statistical results were examined by two authors independently. Five percent samples were randomly selected to repeat, and 100% consistency was obtained.

Cartilage expression of GDF5 in DDH

DDH model in rats were produced as Bo et al [6] described. DDH rats and controls (12 DDH rats vs 12 controls) were sacrificed at the age of 4 weeks and 8 weeks. Hip tissue sample (hip capsule and femoral head cartilage) were obtained from the animal model for histological analysis. For DDH patients (45 to 65 years old) undergoing hip arthroplasty for secondary hip osteoarthritis, hip tissue samples (Hip capsule, femoral head cartilage and joint ligament) were obtained from DDH patients and controls (12 DDH patients vs 12 controls) and total RNA was prepared as we previously reported. Expression of GDF5 in femoral head cartilage were

compared between DDH patients and controls (3 vs 3). Allelic difference of GDF5 expression was demonstrated in DDH patients with different haplotypes. The tissue expression of GDF5 was measured with real-time PCR using gene-specific primers (sequence available upon request) compared to the endogenous control gene Glyceraldehyde -3-phosphate dehydrogenase (GAPDH). For real-time PCR, 1 μL of cDNA was amplified for 40 cycles by SYBR Premix Ex TaqTM II (TaKaRa) in ABI 7900HT mentioned above. Melting curve analysis was done at the end of the reaction to assess the quality of the final PCR products. All samples were analyzed in triplicate using the 2-ΔΔCt method.

Construction of GDF5-pGL3-basic luciferase reporter plasmids

GDF5-pGL3-basic luciferase reporter plasmids for rs143383 and rs143384 were constructed as previously reported.[4] ATDC5 cells were transfected using 500 ng of pGL3 plasmid DNA and 15 ng of pTK-RL Renilla plasmid in combination with Exgen 500 Transfection reagent (Fermentas, Sankt Leon-Rot, Germany). Twenty-four hours after transfection ATDC5 were lysed, using passive lysis buffer (Promega), and the protein extracted and stored at -20◦ C. Lysate sample was mixed with luciferase activating reagent II (Promega) and a 1-s luciferase activity reading was measured using a luminometer (EG&G Berthold, Bad Wildbad, Germany). Stop and Glo (Promega) was added to each sample to measure renilla activity. An empty vector transfection was performed as a control. Each experiment contained six replicates, and was repeated three times, producing a total of 18 data points. A Student’s t-test was performed to assess any significant differences in luciferase activity between the different haplotypes.

Isolation and treatment of BMSC in vitro

Rabbit BMSCs were isolated from rabbit bone marrow aspirates. Briefly, marrow aspirates (20 mL volume) were harvested and immediately transferred into plastic tubes. Isolated BMSCs were expanded in α-MEM containing 10% FBS, 4.5 mg/mL D-glucose,0.1 mM nonessential amino acids, 1 mM sodium pyruvate, 100 mM Hepes buffer, 100 UI/mL penicillin, 100 μg/mL streptomycin, and 0.29 mg/mL L-glutamate. Medium was changed twice a week and BMSCs were used at P2 for the following experiments. In the exogenous GDF5 group, GDF5 (100 ng/ml) was added in the medium for 2 weeks. GDF5 neutralizing peptide was added in some of the cultures according to the protocol. Medium was also changed twice a week. experiments. GDF5 knockdown was conducted with GDF5 siRNAs. GDF5 siRNAs and their negative controls were purchased from GenePharma (Shanghai, China). After validation of inhibition efficacies, a selective GDF5 siRNA (150nM) or negative control was transfected into primary BMSCs cultured. using Lipofectamine 2000 (Invitrogen, Carlsbad, CA, USA) according to the manufacturer’s instructions. GDF5 overexpression was generated with an adenovirus carrying the GDF5 gene (Ad-GDF5) using the AdEasyTM adenoviral vector system (Stratagene, La Jolla, CA) as described previously.[5] We infected the rabbit BMSCs at passage 2 with Ad-GDF5 at a multiplicity of infection of 150 for 24 h. Immunofluorescence staining of chondrogenic marker Col2A1 was conducted to compare the phenotypes of generated chondrocytes in different groups and observed under confocal microscopy (Leica, Japan). The expression of chondrogenesis marker (SOX9, Col2A1) superficial zone chondrocyte markers (GDF5) and deep zone chondrocyte marker (Col10A1) after 4-week incubation was analyzed by real-time polymerase chain reaction (RT-PCR) using an ABI 7300 RT-PCR system (Applied

Biosystems, USA). Four-week-old tissue generated under different conditions was observed and stained with alcian blue for proteoglycan production. The stained images were taken using a light microscope (Leica Microsystems, Germany).

Migration assay of BMSCs with different treatments in vitro

Transwell assay: A transwell culture chamber with a polycarbonate membrane (CoStar, Cambridge, MA), was used to monitor BMSC migration in different treatment groups. Briefly, BMSCs (passage 2) were suspended in α-MEM with 0.2% BSA and 0.5% FCS at a concentration of 2X105 cells/ml. 0.1 ml aliquots of cell suspension were added to the top chamber in 24 well plate. The lower chamber contained 0.6 ml of α-MEM with 0.5% FCS and 0.2% BSA. After the cell seeded, GDF5 was added in the lower chamber Incubation was performed for24h at 37 °C. After 24 hours, cells were fixed with cold methanol after washing with PBS. The upper side was scraped gently to remove the non-motile cells. The cells were staining with crystal violet stain, and examined under a light microscope. The number of migrated cells was scored for six independent fields per transwell filter. The average was used as the cell migration efficiency.

BMSC scratch assay in hydrogel: In order to check the wound healing potential of released GDF5, BMSCs were seeded into 24-well plates pretreated with our composite hydrogel (Table S1) and grown to confluency. After 24 h of serum starvation (1% FBS), lesions were made in the monolayer using cell scraper. Cells were rinsed with PBS, stained with calcein dye and then incubated in different experimental groups (control and 100ng/ml GDF5) for 24 h. Cells were fixed with 4% paraformaldehyde after 24 h and the number of cells which had moved

across the starting scratched lines was measured for both groups.

BMSC migration in the scaffold: To demonstrate the potential of GDF5 for scaffold-based cartilage tissue engineering, BMSCs were seeded into 24-well plates and grown to confluency. The PCL scaffolds were placed atop the monolayer-cultured BMSCs and incubated in different groups (control and 100ng/ml GDF5) for 2 weeks in vitro. After 2 weeks, the scaffolds were fixed with 4% paraformaldehyde after 24 h and cut in half. Migrated Cells in the scaffolds were confirmed by DAPI/Phalloidin staining. Briefly, phalloidin (Thermo Fisher Scientific, USA) was used to stain the F-actin for 1 h and incubated with DAPI (Thermo Fisher Scientific, USA) to stain the nucleus for 5 min in turn. The migration distance was measured for BMSCs in the scaffolds for both groups on confocal microscopy.

Fabrication of GDF-5 conjugated BMSC-laden scaffold for cartilage repair

3d-bioprinting GDF5-conjugated rabbit-derived BMSC-laden hydrogels together with biodegradable polymers was conducted for cartilage construction using organ printing united system (OPUS, *Novaprint*). BMSC suspension (a total of 1×10^7 cells) was loaded into the composite hydrogel. (Table S1) Composite hydrogel as BMSC carrier material is a mixture of gelatin, fibrinogen, HA and glycerol (all purchased from Sigma-Aldrich). In brief, HA was dissolved in DMEM (high glucose) by stirring the solution at 37 °C overnight. Glycerol was added to the solution and stirred for 1 h, which was further shaken after adding gelatin and fibrinogen for 1 h and resulted in final concentrations of gelatin (45 mg/ml), fibrinogen (30 mg/ml), HA (3 mg/ml) and glycerol (10% v/v). Prepared solution was filtered through a 0.45-μm syringe filter and was stored at −20 °C before use. Dynamic shear oscillation measurements

were conducted to characterize the viscoelastic properties of cross-linked composite hydrogel with CSL rheometer (TA Instruments). Storage and loss modulus were recorded in a constant strain mode with a deformation of 0.05 maintained over the frequency range of 0.01-10 Hz (rad/s) at 17 °C. The temperature dependence of the storage and loss modulus was determined by oscillatory shear deformation (dynamic rheological observations) with temperature ranging from 15 to 45 °C (heating rate 1.45 °C min^{-1}) at constant frequency (1 Hz) and constant shear strain (γ=0.05, 1.88 mrad). Before scaffold printing, BMSCs were gently mixed with the composite solution at 37 °C. The supporting material PCL (Mw;43,000~ 50,000, Daigang, Inc., Shandong, China) was used through the melting process. We produced the 3D cartilage structures by placing together cell-laden hydrogel and PCL (~100 μm diameter for hydrogel and ~200 μm diameter for PCL) to construct a composite cartilage scaffold. Briefly, PCL was molten at 60℃ to fabricate the supporting structure for the scaffold while BMSC-laden hydrogel encapsulating PLGA microparticles carrying GDF5 in a different syringe was bio-printed into the microchannels between PCL fibers. During plotting, the needle diameter, layer thickness and speed for PCL printing were kept constant at 200 μm, 200 μm and 180 mm/min, respectively. The fiber spacing was kept constant at 300μm throughout the scaffolds. The scaffolds were plotted in blocks of 4 mm in width and 4mm in height for the cartilage construct. After printing, the printed 3D architectures were cross-linked by the addition of a thrombin solution (20 UI/ml, Sigma-Aldrich) for 30 min at room temperature. After cross-linking, the uncross-linked components (gelatin, HA and glycerol) were washed out with PBS solution for three times, and the PBS solution was exchanged with culture medium. The biomechanical properties of the scaffold after 12-week cultivation in vitro were assessed using

a material-testing machine (Instron 5843, USA). Each sample (1 mm thick) was cut into a rectangular shape. Uniaxial tensile testing was performed on the samples as previously described.[6] The samples were tested to failure at a rate of 0.06 mm/s. Elastic modulus was analyzed from the linear portion of the stress-strain curve. Degradation rate for the composite hydrogel and PCL was assessed in vitro and in vivo. Each PCL scaffold (4 mm × 4mm× 4mm) and composite hydrogel(2cm x 300μm) was immersed into 10 mL of PBS containing 0.1% (w/w) sodium azide, 100 μg/mL streptomycin, and 100 U/mL penicillin at 17 °C. At certain points, the scaffolds and hydrogel were washed with distilled water, dried in vacuum, and weighed. In Vivo Degradation was conducted in nude mice with implantation of the scaffolds and hydrogel subcutaneously on
both sides of the spinal column. After regular times, implanted samples were retrieved with attached tissues and washed with PBS and weighed to calculate weight loss

rhGDF5 was conjugated in PLGA (50:50 PLA/PGA) microspheres (μS) to deliver GDF5 (200ng/ml; PeproTech) in hydrogel as previously described.[7, 8] PLGA μS was fabricated with established double emulsion technique. Briefly, 50μl rhodamine
(2mg/ml) aqueous solution or GDF5 buffer solution (200ng/ml GDF5 in 20mM sodium acetate buffer with pH=6.5) was emulsified into 1ml of 10% PLGA/dichloromethane (DCM) solution. A probe sonicator was used at 15W for 10s in ice bath to form the primary water-in-oil (w/o) emulsion. The w/o emulsion was further mixed with 20ml 1% PVA aqueous solution under sonication to form a water-in-oil-in-water (w/o/w) double emulsion. The solution was then stirred magnetically at room temperature for at least 12h to

evaporate dichloromethane and centrifuged to collect solid microspheres. The retrieved μS was washed with distilled water twice, freeze-dried and stored under vacuum. Morphology of microspheres was examined using Scanning Electric Microscopy (SEM, Philips XL30 FEG). GDF-conjugated μS, with empty μS served as control, were mixed in the BMSC-laden hydrogel (Table S1) respectively and bio-printed into the microchannels between PCL fibers with different syringes. SEM images of PLGA μS were taken, showing the diameter for generated PLGA μS. The printability of the PLGA encapsulated BMSC-laden hydrogel was also shown with a test run. Release kinetics of GDF5 from PLGA μS with different proportions were measured by incubating microspheres (10 mg/ml) encapsulating GDF5 (in phosphate-buffered saline) at 37°C with mild agitation for up to 60 days. Upon centrifugation at 2500 rpm for 5 min, supernatant of the PLGA μS incubation solution was collected. Released GDF5 concentration was measured using enzyme-linked immunosorbent assay (ELISA) kits following the manufacturer's protocols. To validate μS distribution in MSC cell-laden hydrogel, fluorophore-conjugated rhodamine was encapsulated in to PLGA μS and delivered to the hydrogel. At day 7, PLGA rhodamine μS distribution as well as its cell toxicity in the hydrogel was observed under confocal microscope. Cell proliferation through 21 days in the constructs was assessed with AlamarBlue assay kit (DAL1100; Life Technologies) according to the manufacturer's instruction as previously described. [9]

Animal experiments

Ectopic cartilage formation in vivo

The animal experiment protocols were approved by Shanghai Ninth Hospital, medical school of Shanghai Jiao Tong University Ethics Committee and the local Institutional Animal Care and Use Committee (IACUC) and complied with the Guide for the Care and Use of Laboratory Animals published by the National Academy Press (National Institutes of Health Publication No. 85-23, revised 1996). BMSCs or induced cells were suspended at 1 × 107 cells/ml in α-MEM containing 10% FBS. Then, 100 μl of the cell suspension was injected subcutaneously into the dorsal flank of 6-week old female nude mice. Mice were sacrificed after 4 weeks, and the injected sites were dissected from the mice. The samples were fixed in 4% paraformaldehyde, processed, and embedded in paraffin. Serial sections (4-μm thick) of the generated ectopic cartilage were cut through the center of the injection site and stained with alcian blue toluidine blue and safranin-O according to standard protocols. Immunohistochemical staining of chondrocyte marker COL2A1 was conducted according to standard protocols in the generated cartilage tissue sections in different groups. The stained images were taken using a light microscope. GAGs and types II and X collagen were quantitatively assayed (6 vs 6) and normalized to DNA content. GAG production and COL II and X expression was compared among different treatment groups.

Cartilage injury model of rabbit knee joint

Adult male New Zealand white rabbits weighing 3.0–3.5 kg were used. Joint surface injury was performed (n=12) by medial parapatellar arthrotomy as reported.[10] An incision was made to open up the skin over the rabbit knee joint area, followed by an incision along the medial side of the patellar ligament and through the quadriceps muscle to aid patellar dislocation. The

patellar groove was exposed and a full cartilage thickness scratch along the length of the groove was made using a 25 g needle. The patella was then re-located, and the joint capsule and skin were sutured separately. The contralateral leg served as internal uninjured control. rabbits were sacrificed after 8 weeks for further study. Cartilage samples were fixed in 4% paraformaldehyde, processed, and embedded in paraffin. Serial sections of the cartilage were stained with safranin-O according to standard protocols. The twelve operated rabbit knees were then divided into two groups, according to the intensity of safranin-O staining (strong vs weak) in the repaired cartilage. Immunohistochemical staining of GDF5 was conducted according to standard protocols for the repaired cartilage sections in the two groups of different intensity of safranin-O staining. The stained images were taken using a light microscope.

Cartilage defect model in rabbit knee joint

Rabbits were used to examine the cartilage repairing effect of GDF5-conjugated BMSC-laden scaffold in a cartilage defect model in vivo. After the skin incision, a 3-cm medial parapatellar incision was applied and the patella was dislocated. Cylindrical cartilage defects were created at the non-weight-bearing surfaces between the medial and lateral condyles with the size of 4mm wide and 4mm deep, by using electric drill. Rabbits were randomized into three groups (n=6 for each group; two knees of each rabbit were used): GDF-conjugated BMSC-laden scaffold, non-GDF5 BMSC-laden scaffold and the native group with sham surgery. After the operation, rabbits were allowed to move freely in their single cages and fed with standard food and water. Synovial fluid of the operated knee joint was collected at different time points. Intra-articular inflammatory response was quantified by IL-1 concentration measured (n=6 for

each group) using enzyme-linked immunosorbent assay (ELISA) kits following the manufacturer's protocols. 8, 12, and 24 weeks later, rabbits were sacrificed for further study. Serial sections (4-μm thick) were cut sagittally through the center of the operative site and stained with H&E, toluidine blue, Safranin-O & fast green and alcian blue according to standard protocols. Immunohistochemical staining of cartilage markers (aggrecan and Collagen II) was also conducted according to standard protocols in the generated cartilage tissue sections in different groups compared to the native cartilage. The stained images were taken using a light microscope. A modified method was used to evaluate the histological repair of articular cartilage defects.[11] Chondroprotective effects of the scaffolds were examined by evaluating cartilages of the medial FC and TP according to the criteria of the ICRS cartilage lesion classification system and Mankin grading system. .[12]

Statistics

The association between the DDH patients and the control subjects in the stages was tested by SAS software (version 9.2 - SAS Institute, Cary, NC, USA). Bilateral chi square tests were conducted to determine the significance of differences in allelic frequencies and P < 5 x 10-8 was considered to be statistically genome-wide significant. Sample sizes for all other quantitative data were determined by power analysis with one-way ANOVA or two-way ANOVA. All statistical data were expressed as means ± SD. All data analyses were performed using SPSS statistical software (version 15.0; SPSS Inc.). Values of $P < 0.05$ were considered statistically significant.

References

1. Sun Y, Wang C, Hao Z, Dai J, Chen D, Xu Z, et al. A common variant of ubiquinol-cytochrome c reductase complex is associated with DDH. PLoS One. 2015; 10: e0120212.

2. Hatzikotoulas K, Roposch A, Consortium DDHCC, Shah KM, Clark MJ, Bratherton S, et al. Genome-wide association study of developmental dysplasia of the hip identifies an association with GDF5. Commun Biol. 2018; 1: 56.

3. Rouault K, Scotet V, Autret S, Gaucher F, Dubrana F, Tanguy D, et al. Evidence of association between GDF5 polymorphisms and congenital dislocation of the hip in a Caucasian population. Osteoarthritis and cartilage. 2010; 18: 1144-9.

4. Dodd AW, Syddall CM, Loughlin J. A rare variant in the osteoarthritis-associated locus GDF5 is functional and reveals a site that can be manipulated to modulate GDF5 expression. Eur J Hum Genet. 2013; 21: 517-21.

5. Feng G, Wan Y, Balian G, Laurencin CT, Li X. Adenovirus-mediated expression of growth and differentiation factor-5 promotes chondrogenesis of adipose stem cells. Growth Factors. 2008; 26: 132-42.

6. Patel JM, Merriam AR, Culp BM, Gatt CJ, Jr., Dunn MG. One-Year Outcomes of Total Meniscus Reconstruction Using a Novel Fiber-Reinforced Scaffold in an Ovine Model. The American journal of sports medicine. 2016; 44: 898-907.

7. Morille M, Toupet K, Montero-Menei CN, Jorgensen C, Noel D. PLGA-based microcarriers induce mesenchymal stem cell chondrogenesis and stimulate cartilage repair in osteoarthritis. Biomaterials. 2016; 88: 60-9.

8. Wei G, Jin Q, Giannobile WV, Ma PX. Nano-fibrous scaffold for controlled delivery of

recombinant human PDGF-BB. J Control Release. 2006; 112: 103-10.

9. Kang HW, Lee SJ, Ko IK, Kengla C, Yoo JJ, Atala A. A 3D bioprinting system to produce human-scale tissue constructs with structural integrity. Nat Biotechnol. 2016; 34: 312-9.

10. Kiener HP, Watts GF, Cui Y, Wright J, Thornhill TS, Skold M, et al. Synovial fibroblasts self-direct multicellular lining architecture and synthetic function in three-dimensional organ culture. Arthritis Rheum. 2010; 62: 742-52.

11. Wakitani S, Okabe T, Horibe S, Mitsuoka T, Saito M, Koyama T, et al. Safety of autologous bone marrow-derived mesenchymal stem cell transplantation for cartilage repair in 41 patients with 45 joints followed for up to 11 years and 5 months. J Tissue Eng Regen Med. 2011; 5: 146-50.

12. Brittberg M, Winalski CS. Evaluation of cartilage injuries and repair. The Journal of bone and joint surgery American volume. 2003; 85-A Suppl 2: 58-69.

Table S1. Meta-analysis incorporating rs143383 and rs143384 of GDF5 from GWAS results and replication study results in different populations

	Chinese GWAS (386 cases, 558 controls)			U.K. GWAS (770 cases, 3364 controls)			Chinese Replication (218 cases, 360 controls)			France Replication (239 cases, 239 controls)			Meta-analysis of GWAS + Replications combined (1613 cases, 4521 controls)		
**SNP**	**Case MAF**	**OR**	**P Value**	**Case MAF**	**OR**	**P Value**	**Case MAF**	**OR**	**P Value**	**Case MAF**	**OR**	**P Value**	**Case MAF**	**OR**	**P Value**
**rs143384**	**0.205**	**0.73(0.59-0.92)**	**0.006**	**0315**	**0.64(0.57-0.72)**	**1.72E-14**	**0.202**	**0.72 (0.54-0.96)**	**0.02**	**0.341**	**0.66 (0.51-0.85)**	**0.002**	**0.278**	**0.66 (0.60-0.73)**	**8.02E-30**
**rs143383**	**0.201**	**0.71(0.57-0.88)**	**0.002**	**0.286**	**0.66(0.59-0.75)**	**1.29E-11**	**0.195**	**0.67(0.50-0.90)**	**0.007**	**0.316**	**0.73 (0.56-0.96)**	**0.02**	**0.258**	**0.68 (0.62-0.75)**	**2.68E-23**

Table S2. Meta-analysis of other potential signals surrounding GDF5 gene in the discovering stage of both GWAS to retrieve significant associations for the loci.

	Chinese GWAS (386 cases, 558 controls)			U.K. GWAS (770 cases, 3364 controls)			Meta-analysis GWAS (1156 cases, 3922 controls)		
rs# Chr position	Case MAF	OR (95% CI)	P Value	Case MAF	OR (95% CI)	P Value	OR (95% CI)	P Value	
rs6088765 20:33799280	0.32	0.77(0.63-0.93)	0.0075	0.359	0.75(0.67-0.84)	7.5E-07	0.75(0.68-0.83)	**1.9E-08**	Downstream
rs12479765 20:33825378	0.088	0.73(0.53-0.99)	0.042	0.133	0.70(0.60-0.83)	1.6E-05	0.71(0.61-0.82)	**1.9E-06**	Downstream
rs878639 20:33894463	0.187	0.57(0.46-0.72)	1.4E-06	0.298	0.68(0.61-0.77)	2.4E-10	0.65(0.57-0.73)	**5.8E-15**	Downstream
rs6088792 20:33909784	0.088	0.60(0.44-0.81)	8.1E-04	0.237	0.72(0.63-0.82)	6.2E-07	0.70(0.62-0.79)	**3.7E-09**	Downstream
rs6060373 20:33914208	0.185	0.58(0.47-0.73)	1.1E-06	0.299	0.69(0.61-0.77)	3.6E-10	0.65(0.58-0.72)	**6.7E-15**	Downstream
rs4911178 20:33952620	0.188	0.6（0.49-0.74）	2.3E-06	0.294	0.67(0.59-0.76)	2.7E-11	0.65(0.58-0.72)	**1.1E-15**	Downstream
rs4911494 20:33971914	0.187	0.58(0.47-0.72)	2.1E-06	0.294	0.67(0.59-0.75)	2.9E-11	0.65(0.58-0.72)	**8.9E-16**	Downstream
rs6088813 20:33975181	0.187	0.58(0.47-0.72)	1.7E-06	0.294	0.67(0.59-0.76)	2.7E-11	0.65(0.58-0.72)	**8.9E-16**	Downstream
rs6087704 20:34001058	0.187	0.58(0.48-0.73)	1.7E-06	0.294	0.67(0.59-0.76)	1.3E-11	0.65(0.58-0.72)	**6.7E-16**	Downstream
rs6087705 20:34001250	0.187	0.58(0.48-0.73)	2.1E-06	0.294	0.67(0.69-0.76)	3.7E-11	0.65(0.58-0.72)	**8.9E-16**	Downstream
rs1570841 20:34048161	0.22	0.66(0.53-0.81)	1.2E-04	0.181	0.70(0.61-0.81)	8.1E-07	0.69(0.61-0.77)	**4.4E-10**	Upstream
rs4911507 20:34075998	0.174	0.62(0.49-0.78)	5.1E-05	0.133	0.71(0.60-0.83)	2.4E-05	0.68(0.60-0.77)	**7.3E-09**	Upstream
rs3748433 20:34090519	0.134	0.65(0.50-0.83)	8.2E-04	0.06	0.65(0.52-0.82)	2.3E-04	0.65(0.55-0.77)	**6.4E-07**	Upstream
rs2236164 20:34097353	0.219	0.64(0.52-0.79)	4.5E-05	0.181	0.70(0.61-0.81)	8.1E-07	0.68(0.61-0.77)	**1.9E-10**	Upstream
rs224419 20:34143092	0.17	0.71(0.56-0.89)	0.0036	0.181	0.73(0.63-0.84)	1.1E-05	0.72(0.64-0.81)	**1.4E-07**	Upstream

Table S3 Preparation of the cell-laden composite hydrogels for 3D-bioprinted meniscus constructs

Gelatin	Fibrinogen	HA	Glycerol	Cell type & density
45 mg/ml	30 mg/ml	3 mg/ml	10% v/v	BMSCs, 1 × 10^7 cells/ml

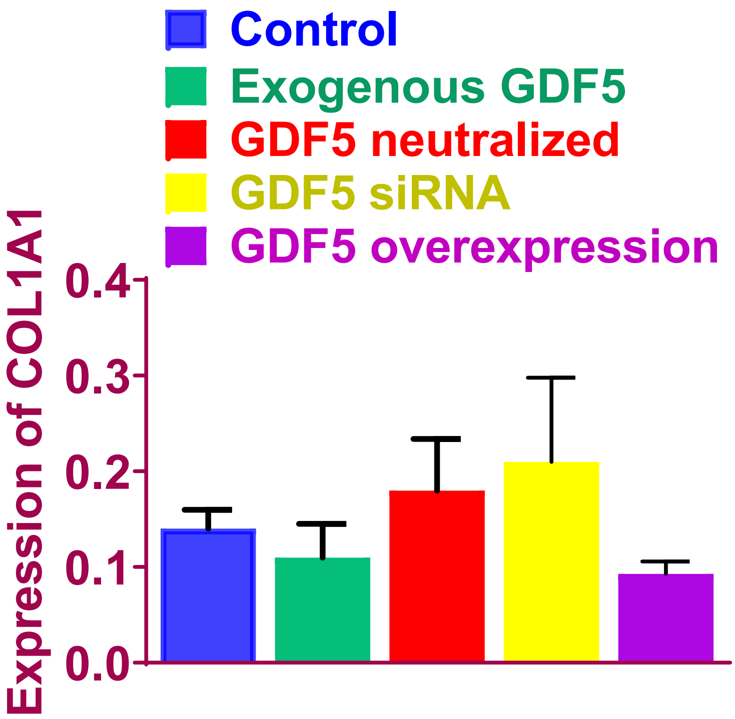

Figure S1. Expression of COL1A1 in different treatment groups. Expression of COL1A1 in different treatment groups (n=6 for each) were verified using RT-PCR. *P < 0.05 between control group and other groups
